# 953. Online Medical Education Improves Knowledge of Monoclonal Antibody Treatment for COVID-19 Among Physicians

**DOI:** 10.1093/ofid/ofab466.1148

**Published:** 2021-12-04

**Authors:** Allison Armagan, Maria B Uravich

**Affiliations:** 1 Medscape, New York, New York; 2 Medscape Education, New York, New York

## Abstract

**Background:**

Treatments aimed at patients with mild to moderate COVID-19 offer an opportunity to improve rates of hospitalizations and progression to severe disease. The aim of this study was to assess the educational impact of a series of continuing medical education (CME) activities on the knowledge, competence, and confidence of primary care (PCP), infectious disease (ID), and ER/critical care physicians regarding the management of COVID-19 with monoclonal antibody (mAb) therapy.

**Methods:**

The educational series consisted of 9 online, CME activities in multiple formats. At the individual activity level, educational effect was assessed with a repeated pairs pre-/post-assessment study including a 3-item, multiple choice, knowledge/competence questionnaire and one confidence assessment question, with each participant serving as his/her own control. To assess changes in knowledge, competence, and confidence data from all clinicians who completed both pre- and post-questions were aggregated across activities and stratified by learning themes. McNemar’s test (P< .05) assessed educational effect. Data were collected from 12/20 to 5/21.

**Results:**

To date, the 9 activities have reached over 24,000 physicians. Selected improvements in knowledge and competence measured as relative % change in correct responses pre/post education across the learning themes are reported here. (i) 45% improvement in PCPs and a 31% improvement in ID specialists’ knowledge/competence in identifying patients who would benefit from mAbs (*P* < .01). (ii) 83% improvement in PCPs and a 42% improvement in ID specialists’ confidence in identifying patients who would benefit from mAbs (*P* < .001). (iii) 15% improvement in ID specialists’ knowledge/competence on the clinical data on mAbs for COVID-19 (*P* < .001). (iv) 32% improvement in PCPs knowledge/competence in understanding the mechanism of action (MOA) of mAbs for COVID-19 (*P* < .001)

**Conclusion:**

This series of online, CME-certified educational activities delivered in multiple formats resulted in significant improvements in knowledge and competence regarding the management of patients with mild to moderate COVID-19. This analysis also uncovered remaining educational gaps; 55% of content related to identifying patients who would benefit from mAbs was not retained post-education.

**Disclosures:**

**All Authors**: No reported disclosures

